# Clinical Use of Acid Suppressants and Risk of Dementia in the Elderly: A Pharmaco-Epidemiological Cohort Study

**DOI:** 10.3390/ijerph17218271

**Published:** 2020-11-09

**Authors:** Liang-Yu Chen, Huey-Juan Lin, Wen-Tung Wu, Yong-Chen Chen, Cheng-Li Chen, Jing Kao, San-Lin You, Yu-Ching Chou, Chien-An Sun

**Affiliations:** 1 Department of Pharmacy Practice, Tri-Service General Hospital, National Defense Medical Center, Taipei City 114, Taiwan; rainy1316@gmail.com (L.-Y.C.); allen541312@gmail.com (W.-T.W.); 2 School of Public Health, National Defense Medical Center, Taipei City 114, Taiwan; jor820401@gmail.com (C.-L.C.); kosukeluvu@gmail.com (J.K.); 3 Department of Neurology, Chi-Mei Medical Center, Tainan City 710, Taiwan; huikuanlin@gmail.com; 4 Department of Medicine, College of Medicine, Fu-Jen Catholic University, New Taipei City 242, Taiwan; yongchenchen0824@gmail.com (Y.-C.C.); yousanlin@gmail.com (S.-L.Y.); 5 Big Data Research Center, College of Medicine, Fu-Jen Catholic University, New Taipei City 242, Taiwan; 6 Department of Public Health, College of Medicine, Fu-Jen Catholic University, New Taipei City 242, Taiwan

**Keywords:** dementia, histamine 2 receptor antagonist, proton pump inhibitors, retrospective cohort study

## Abstract

Background: Results of studies regarding the potential link between acid suppressant use and dementia risk are inconsistent. This study aimed to evaluate the association of cumulative exposure to histamine 2 receptor antagonists (H2RAs) and proton pump inhibitors (PPIs) with dementia risk in an Asian older cohort aged ≥65 years. Methods: Patients initiating H2RA (the H2RA user cohort, *n* = 21,449) or PPI (the PPI user cohort, *n* = 6584) and those without prescription for H2RA (the H2RA non-user cohort, *n* = 21,449) or PPI (the PPI non-user cohort, *n* = 6584) between 1 January 2000 and 31 December 2005 without a prior history of dementia were identified from Taiwan’s National Health Insurance Research Database (NHIRD). The outcome of interest was all-cause dementia. Patients’ exposure to H2RAs or PPIs was followed-up from dates of initial prescription to the earliest outcome of incident dementia, death, or the end of 2013. Potential associations between acid suppressant use and dementia risk were analyzed using time-dependent Cox regression estimated hazard ratios (HRs) and 95% confidence intervals (CIs). Results: After mutual adjustment for H2RA and PPI use and other potential confounders, patients with H2RA use had significantly higher risk of developing dementia as compared to those not treated with H2RAs (adjusted HR, 1.84; 95% CI, 1.49–2.20). Likewise, PPI users had significantly elevated risk of dementia compared to PPI non-users (adjusted HR, 1.42; 95% CI, 1.07–1.84). Conclusions: Our results indicate that exposures to H2RAs and PPIs are associated with increased dementia risk.

## 1. Introduction

A dramatic increase in the prevalence of comorbid chronic disease and exposure to multiple medications has accompanied the ageing of the population worldwide [1,2]. The comorbidities associated with ageing have led to a higher rate of chronic medication use in elderly populations [1]. Growing evidence suggests that several commonly used medications in elderly populations may be associated with diverse cognitive outcomes including cognitive impairment or dementia [3,4]. Dementia, a syndrome of cognitive decline and a major cause of disability in older age, has become a global public health priority in the context of population ageing [5,6]. The number of people affected by dementia is expected to increase to 131 million in 2050 [6]. Besides the substantial burden on patients and their families, dementia also affects health care systems worldwide.

Histamine 2 receptor antagonists (H2RAs) and proton pump inhibitors (PPIs) are among the most widely used pharmacological treatment of various gastrointestinal disorders in older population [7]. Effective pharmacologic inhibition of gastric acid secretion began with the introduction of H2RAs in the 1970s, which greatly improved clinical outcomes. The development of PPIs further improved suppression of gastric acid secretion and ensured very high healing rates for duodenal and gastric ulcers [8]. Although PPIs have a stronger acid inhibition property than H2RAs, patients using H2RAs are at a lower risk of pneumonia and *Clostridium difficile* infection than PPI users [8]. Thus, even with the advent of PPIs, H2RAs remain widely used. Histamine is a neuroactive amine and plays an important role in cognitive function [9]. Several epidemiological studies have examined the effect of H2RA use on diverse cognitive outcomes, including cognitive impairment [10], Alzheimer’s disease (AD) [11,12,13,14], and dementia [15,16], with mixed results. Several [11,12] but not all [13] cross-sectional studies found an association between use of H2RAs and lower risk of AD. However, two follow-up studies did not confirm this association [10,14]. Another follow-up study reported that H2RA use was associated with an increased risk of AD or cognitive decline in African Americans [16]. In addition, proton pump inhibitors are potent suppressors of gastric acid secretion and the use of PPIs has increased tremendously, especially among the elderly [17,18]. Observational data suggests that PPI use might drive risk of cognitive dysfunction. Lam et al. reported a significant association between PPI use and vitamin B_12_ deficiency in a population-based study [7]. Vitamin B_12_ deficiency has been shown to be associated with cognitive decline [19]. However, the results of epidemiological studies on the association of PPI use with cognitive impairment, such as dementia, have shown inconsistent conclusions [20,21,22,23,24,25,26,27,28,29,30,31]. Several cohort studies indicated that PPIs were associated with an increased risk of dementia [20,21,22,23], whereas a case-control study reported that PPIs reduced the risk of dementia [31]. More recent studies point towards a null association between PPI use and dementia risk [25,26,27,28,29,30]. Given these discrepant results and the importance of maintaining adequate cognitive function in elders, the objectives of the present study were to examine the longitudinal associations of the use of acid suppressants, including H2RAs and PPIs, with incident dementia in a cohort of Asian elderly population, given the shared clinical indications between H2RAs and PPIs.

## 2. Materials and Methods

### 2.1. Data Source

The present study was a population-based retrospective cohort study using the Taiwan National Health Insurance (NHI) claims database-National Health Insurance Research Database (NHIRD). The NHI has been a single-payer, universal, compulsory health care system for nearly all 23.7 million residents in Taiwan since March 1995. The NHIRD contains comprehensive health care information, including demographic data of insured individuals, outpatient visits, hospital admission, disease diagnostic codes, and prescription details. The diagnostic codes used in the NHIRD follow the International Classification of Diseases, 9th Revision, Clinical Modification (ICD-9-CM) (Medicode, Salt Lake City, UT, USA). NHIRD had been used for high quality epidemiological studies [32,33,34] and has been demonstrated to show good validity of data on diagnoses and prescriptions [35,36,37]. The data for this study was obtained from the Longitudinal Health Insurance Database (LHID 2000). LHID 2000 is a cohort dataset of original medical claims data that includes one million beneficiaries systematically randomly sampled from the registry of NHIRD. Although the dataset included medical records from the start of 1996, the data for the first few years were incomplete. Accordingly, we only analyzed the longitudinal data between the start of 2000 and the end of 2013. There was no significant difference in the distributions of age, sex, and health care costs between the individuals in LHID 2000 and all enrollees in NHIRD [38]. These data files are de-identified by scrambling the identification codes of all beneficiaries and information obtained from the databases was entirely anonymous. Since the dataset was released for research purposes and included only scrambled information on insured individuals, the requirement for written or verbal consent from patients for study was waived, while the protocol of the present study has been approved by the Institutional Review Board of Fu-Jen Catholic University (FJU-IRB No: C104014).

### 2.2. Participants

**Figure 1:**
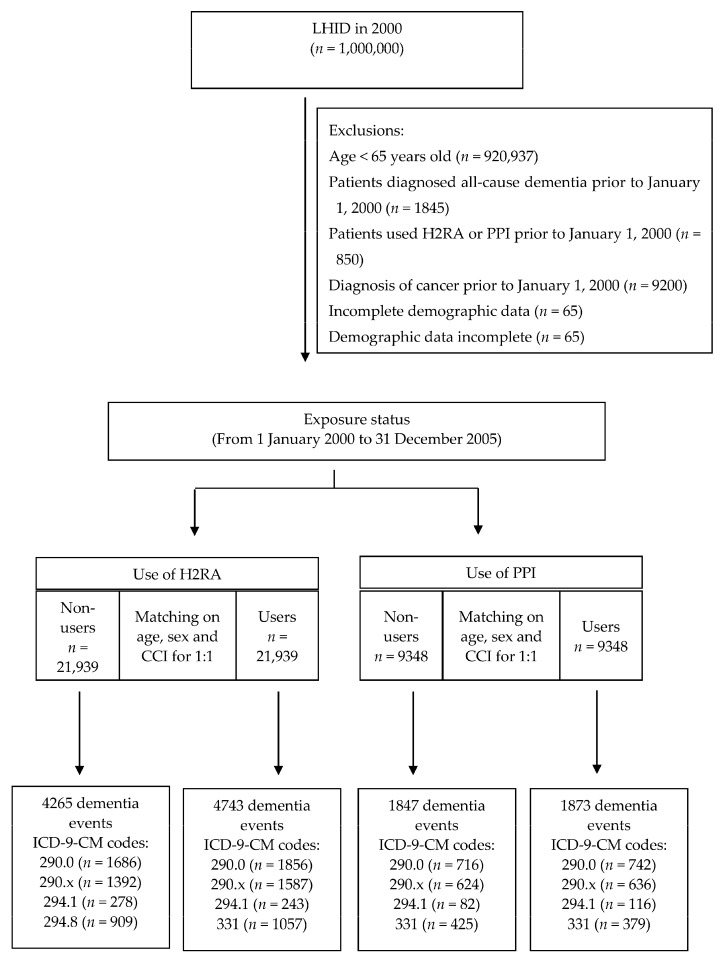
Study flowchart. x = 1 or 2 or 3 or 4. LHID, Longitudinal Health Insurance Database; H2RA, histamine 2 receptor antagonist; PPI, proton pump inhibitors; CCI, Charlson comorbidity index; ICD-9-CM, International Classification of Diseases, 9th Revision, Clinical Modification.

Patients aged ≥65 years were included because dementia is most prevalent in this age group [39]. The primary exposure examined was cumulative use of H2RAs or PPIs. The outpatient pharmacy prescription database cross-linkage and drug data files were used to determine the usage of H2RAs and PPIs in individual patients. Patients who had initially received H2RAs between 1 January 2000 and 31 December 2005 were identified for the H2RA cohort (H2RA users) and were compared with a comparison cohort comprised of patients who had never been treated with H2RAs (hereafter, non-H2RA users). Likewise, patients who had initially received PPIs between 1 January 2000 and 31 December 2005 were identified for the PPI cohort (PPI users) and were compared with a comparison cohort comprised of patients who had never been treated with PPIs (hereafter, non-PPI users). The date of initial prescription of H2RAs or PPIs for each patient was assigned as their index date. Initiation was defined as being free from any H2RA or PPI therapy for 12 months prior to the first prescription. We applied frequency matching at a ratio of 1:1 for the H2RA users’ cohort to the matched H2RA non-users’ cohort and for the PPI users’ cohort to the matched PPI non-users’ cohort with matching for sex, age, index year, and Charlson comorbidity index (CCI), which represent the burden of comorbidities at baseline [40]. To minimize the effect of acid suppressant use prior to the study period (prevalent user bias) [41], we excluded patients who were prescribed H2RAs or PPIs prior to the index date (*n* = 850). In addition, patients were also excluded if they were aged less than 65 years old (*n* = 920,937), had all-cause dementia diagnosed before 2000 (*n* = 1845), were ever diagnosed with cancer (ICD-9-CM codes 140–239) before 2000 (*n* = 9200), or had incomplete demographic data (*n* = 65). A total of 21,939 patients with H2RA use, 21,939 patients without use of H2RA, 9348 patients with PPI use, and 9348 patients without use of PPI were observed until they were diagnosed with all-cause dementia, died (as indicated by disenrollment from the NHI), or until 31 December 2013, whichever came first (Figure 1).

### 2.3. Exposure Measurement of Studied Medications

The main exposure of interest was clinical use of H2RBs, including cimetidine, ranitidine, famotidine, and roxatidine (the anatomic therapeutic chemical (ATC) codes A02BA01-A02BA03 and A02BA05; nizatidine was not available in Taiwan), or PPIs, including omeprazole, pantoprazole, lansoprazole, esomeprazole, and rabeprazole, supply days, and total number of pills dispensed from the outpatient pharmacy prescription database. Cumulative dosage of H2RB or PPI use during the study period was calculated and presented as the defined daily dose (DDD) by the following formula: (total amount of drug)/(amount of drug in a DDD) = number of DDDs [42]. Cumulative DDD (cDDD) was estimated as the sum of dispensed DDDs of H2RAs or PPIs from January 1 2000 to the date of a diagnosis of all-cause dementia or until the end of the study (31 December 2013). Dosage categories of H2RA or PPI use were classified based on quartile distribution of cDDD among H2RA users and PPI users, respectively. All exposure measures of studied acid suppressants were lagged 1 year to avoid consideration of H2RA or PPI use that might have been influenced by the onset of dementia.

### 2.4. Ascertainment of Dementia

The primary clinical outcome was the incidence of all-cause dementia. We determined patients with dementia as having primary diagnosis of the ICD-9-CM codes of 290.0, 290.1, 290.2, 290.3, 290.4, 294.1, and 331.0. In Taiwan, a board-certified psychiatrist or neurologist primarily confirmed the diagnosis of dementia based on the diagnostic criteria of the Diagnostic and Statistical Manual of Mental Disorders, Fourth Edition. In order to identify patients with dementia with sufficient accuracy, all dementia cases had at least three records of outpatient visits or one admission diagnosis.

### 2.5. Covariate Ascertainment and Adjustment

Potential confounders included sex, age, drug use, and baseline comorbidities. Inpatient and outpatient files from the year 2000 were used to ascertain whether subjects had comorbidities, including hypertension (ICD-9-CM codes 401.1, 401.9, 402.10, 402.90, 404.10, 404.90, 405.1, and 405.9), diabetes mellitus (ICD-9-CM code:250), hyperlipidemia (ICD-9-CM code:272.x), coronary artery disease (ICD-9-CM codes:410–414), stroke (ICD-9-CM codes 430–438), and depression (ICD-9-CM codes 296.2, 296.3, 296.82, 300.4, and 311). Comorbidities were defined in a patient if he or she was diagnosed for any of the aforementioned diseases on at least two outpatient claims or one inpatient claim from the year 2000. The prevalence of comorbidities was characterized using the CCI, which was derived from the ICD-9-CM codes in the claims database. The CCI score, a widely accepted measure for risk adjustment in administrative claims data sets, was calculated using the sum of the weighted scores of all aforementioned comorbidities [40,43]. In addition, we included use of co-medications in the multivariable regression models as potential confounders if they could potentially accelerate or reduce inflammation or cognitive function. These included nonsteroidal anti-inflammatory drugs (NSAIDs), anti-hypertensives, anti-diabetic agents, statins, aspirin, and anti-depressants. Exposure to these medications was defined as having a prescription for one of them from the year 2000 to the date of diagnosis of all-cause dementia, death, or the end of the study period, whichever occurred first.

### 2.6. Statistical Analysis

Chi-square and t tests were used to evaluate the difference in distributions of categorical and continuous variables between the H2RA users cohort vs. the H2RA non-users cohort and the PPI users cohort vs. PPI non-users cohort, respectively. Because the exposure in this observational cohort is time-dependent, the Cox regression model with time-dependent covariates was used to determine the independent and combined effects of exposure to H2RAs or PPIs on incident dementia risk. The results were presented as hazard ratios (HRs) and 95% confidence intervals (CIs). In this model, patients treated with H2RAs or PPIs were defined as the exposure group, and patients would be switched to the non-exposure group when they stopped H2RA or PPI treatment during the study period between 1 January 2000 and 31 December 2005. Several covariates including sex, age, index year, the CCI score, and use of co-medications were adopted as potential confounders in the multivariable Cox regression model. Of note, there are collinearity issues between comorbidities and use of co-medications. Accordingly, anti-hypertensives for hypertension, anti-diabetic agents for diabetes mellitus, statins for hyperlipidemia, and anti-depressants for depression were not included in the multivariable Cox regression model to avoid multicollinearity. In addition, to decrease the potential detection bias, the number of annual outpatient visits, due mainly to either follow-up or continuous treatment, was also included as a potential confounder in the regression model. The dose-response relationship between cumulative exposure to H2RAs or PPIs and risk of dementia after adjustment for potential confounders was examined for statistical significance with a test for trend. All statistical tests were two-sided, and an α level of 0.05 was considered statistically significant. All data analyses were performed using SAS software, version 9.4 (SAS Institute, Cary, NC, USA).

## 3. Results

Table 1 shows distribution of baseline characteristics among participants according to acid suppressant use. There was no difference in the distributions of sex, age, and the CCI score between H2RA users and PPI users cohorts and their respective comparison cohorts, due to the matching scheme. As expected, the prevalence rates of gastric ulcer and duodenal ulcer were significantly higher in both H2RA and PPI users’ cohorts than those in the comparison cohorts. In addition, the patients receiving H2RA or PPI treatment had more comorbidities, including hypertension, diabetes mellitus, hyperlipidemia, coronary artery disease, stroke, and depression, than patients without treatment of H2RA or PPI. In addition, patients’ exposure to H2RA or PPI had more medication use, including NSAIDs, anti-hypertensives, anti-diabetic agents, statins, aspirin, and anti-depressants, than those without use of H2RA or PPI. Further, H2RA users or PPI users tended to have a significantly higher frequency of annual outpatient visits as compared to those who had never used H2RA or PPI.

In the present study, the incidence rates of dementia among H2RA users, H2RA non-users, PPI users, and PPI non-users were 22.71, 19.69, 24.28, and 20.65 per 1000 person-years, respectively. After mutual adjustment for H2RA and PPI use and other potential confounders, patients treated with H2RA had significantly higher risk of developing dementia as compared with those not treated with H2RAs (adjusted HR, 1.84; 95% CI, 1.49–2.20). Likewise, PPI users had a significantly elevated risk of dementia than PPI non-users (adjusted HR, 1.42; 95% CI, 1.07–1.84). Furthermore, we found a significant association between cumulative H2RA or PPI use and risk of dementia (*p*_trend_ < 0.001) (Table 2).

Table 3 presents results of stratified analyses of the risk of dementia associated with use of acid suppressants. Among PPI users, there was no statistically significant association between H2RA use and risk of dementia (adjusted HR, 0.92; 95% CI, 0.85–1.06). However, a statistically significant association of dementia risk associated with the use of H2RAs was apparent among PPI non-users (adjusted HR, 1.86; 95% CI, 1.37–2.02). Likewise, there was no statistically significant association between PPI use and risk of dementia among H2RA users (adjusted HR, 0.76; 95% CI, 0.61–1.83). However, a statistically significant association of dementia risk associated with the use of PPIs was noted among H2RA non-users (adjusted HR, 1.23; 95% CI, 1.08–2.59).

Table 4 further shows the risk of dementia associated with acid suppressant combination. Statistically significant association was found in H2RA-only users (adjusted HR, 1.85; 95% CI, 1.26–2.16) and PPI-only users (adjusted HR, 1.64; 95% CI, 1.14–1.92). However, the association was attenuated with concomitant use of H2RAs and PPIs (adjusted HR, 1.23; 95% CI, 1.07–1.98).

## 4. Discussion

This nationwide population-based longitudinal study of persons aged 65 years and older revealed a significantly increased risk of dementia associated with use of acid suppressants. Specifically, use of H2RAs or PPIs was independently associated with an elevated risk of dementia. Our findings support a detrimental impact of H2RAs and PPIs on the risk of dementia.

It has been noted that H2RAs are one of the most widely used pharmacological therapies for various gastrointestinal disorders in older adults [1,2]. Histamine is an excitatory neurotransmitter and plays an important role in cognitive function [9]. H2RA agents have been shown to have serum anticholinergic activity which could contribute to cognitive impairment [44]. However, epidemiologic research on the effect of H2RA use on the cognitive function in elderly populations has produced inconsistent findings [10,11,12,13,14,15,16,17]. A number of cross-sectional analyses reported null or inverse associations between H2RA use and dementia risk [11,13,14], whereas several cohort studies identified that use of H2RAs was associated with an increased risk of cognitive impairment or dementia [10,16,17]. The findings of the present study are in agreement with prior longitudinal studies that the use of H2RA was a risk factor for subsequent cognitive decline or dementia [10,16,17]. Similarly, the results of epidemiological studies on the association of PPI use with dementia risk have been inconclusive [20,21,22,23,24,25,26,27,28,29,30,31]. Several epidemiological studies reported a detrimental impact of PPIs in increasing the risk of dementia and Alzheimer’s disease [20,21,22,23]. However, a number of epidemiological studies [28,29] and meta-analyses [25,27,30] point toward a null association between PPI use and dementia risk. Of note, a case-control study on risk factors of dementia observed that PPIs were associated with a decreased risk of developing dementia [31]. The results of the current study are consistent with previous findings from cohort studies showing a detrimental effect of PPI use on the risk of dementia [20,21,23]. The reasons for the conflicting findings between studies are not readily apparent. The sources of the discrepancies between studies might be due to methodological issues such as different studied populations (cognitively normal subjects [11,13,17,23,28,29] vs. subjects at high risk for dementia [10,12,14,15,16,20,21,24,26,31], the study design used (cross-sectional [13], case-control [24,31] or longitudinal studies [10,12,14,15,16,17,20,21,23,26,28,29], and the adjustment for potential confounders between studies. In addition, although researchers attempted to increase the reliability of drug exposure assessment data in previous studies, patient-reported drug use [4,5,6,7,8,9,10,11,16,20,26,28,31] remained a critical flaw given that cognition was examined as outcome. Further, it is noted that failure of prior studies to control for PPIs in examining the association between exposure to H2RA and dementia risk and vice versa could have produced varying results depending on the mix of medications in the polypharmacy category.

In the present study, a significant association between H2RA use and risk of dementia was observed among PPI non-users. Likewise, a significant relationship of dementia risk with PPI use was noted among H2RA non-users (Table 3). In addition, concomitant use of PPIs and H2RAs did not synergistically increase the risk of dementia as compared with the use of either PPIs alone or H2RAs alone (Table 4). It has been demonstrated that histamine released from the enterochromaffin-like cells binds to receptors on the parietal cells and leads to increased cyclic adenosine monophosphate, and then proton pump activation [45]. Accordingly, H2RAs could decrease the activation of proton pumps and therefore should interfere with the action of PPIs. Further studies are needed to confirm our study results.

The study had a number of strengths. The present study is a nationwide cohort study based on Taiwan’s NHIRD, which contains claims data from Taiwan’s compulsory and universal health care system which has high coverage rate in Taiwan. This allowed us to perform the analysis in a real-life setting in an unselected patient population. In addition, the diagnoses of dementia in NHIRD were based on ICD-9-CM codes and were determined by relevant specialists and physicians, according to standard clinical criteria. The data on the diagnoses of dementia can thus be considered reliable. Moreover, patient loss-to-follow-up was avoided due to a high coverage rate of NHIRD and recall bias was minimized because of use of the pharmacy prescription database.

Despite the strength of our large-scale population-based cohort study, the results of the present study need to be interpreted within the context of some limitations. Given that there is no commonly prescribed comparable alternative intervention that might serve as a natural control group for PPI or H2RA exposure, confounding by indication might play a role in studying associations between PPI and H2RA use and dementia risk. In addition, studies that are based on insurance claims or other third-party data are often flawed because the information on confounders contained in insurance data is often limited [46]. Accordingly, unmeasured or residual confounding, such as ApoE4 allele status and family history of the disease, could introduce bias in our estimates. It has been noted that non-judicious PPI or H2RA prescription is especially frequent among the elderly [47]. Thus, elderly people who have frequent use of health care systems are at increased risk for both PPI or H2RA prescription and dementia diagnosis. This bias may not be completely mitigated by adjustment for comorbidities or polypharmacy. Further, information with respect to patient adherence to medications or self-paid medications is not available in NHIRD. Non-adherence would most likely result in non-differential misclassification of the exposure, which would lead to underestimation of the actual risk.

## 5. Conclusions

In conclusion, results from this nationwide cohort study of Taiwanese insurance claims data do support a detrimental impact of PPIs and H2RAs on the risk of dementia. Caution should be exercised until randomized clinical trials can elucidate the relationship between acid suppressant use and dementia risk.

## Figures and Tables

**Table 1:** Baseline characteristics of cohort participants by acid suppressant exposure.

Variable	H2RA			PPI		
	Users No. (%)	Non-Users No. (%)	*p* Value	Users No. (%)	Non-Users No. (%)	*p* Value
No. of subjects	21,939	21,939		9348	9348	
Sex			1.000			1.000
Men	10,770 (49.1)	10,770 (49.1)		5537 (59.2)	5537 (59.2)	
Women	11,169 (50.9)	11,169 (50.9)		3811 (40.8)	3811 (40.8)	
Age (years)			1.000			1.000
65–69	7726 (35.2)	7726 (35.2)		2593 (27.7)	2593 (27.7)	
70–79	10,822 (49.3)	10,822 (49.3)		4657 (49.8)	4657 (49.8)	
≥80	3391 (15.5)	3391 (15.5)		2098 (22.4)	2098 (22.4)	
Main indications						
Gastric ulcer	4707 (21.5)	1739 (7.9)	<0.001	3504 (37.5)	737 (7.9)	<0.001
Duodenal ulcer	2672 (12.2)	926 (4.2)	<0.001	2195 (23.5)	398 (4.3)	<0.001
GERD	232 (1.1)	161 (0.7)	<0.001	115 (1.2)	63 (0.7)	<0.001
Comorbidities						
Hypertension	14,420 (65.7)	13,296 (60.6)	<0.001	6047 (64.7)	5642 (60.4)	<0.001
Diabetes mellitus	6134 (28.0)	5483 (24.9)	<0.001	2879 (30.8)	2547 (27.2)	<0.001
Hyperlipidemia	3169 (14.4)	2919 (13.3)	0.005	1215 (13.0)	1022 (10.9)	<0.001
CAD	8752 (39.9)	8139 (37.1)	<0.001	3840 (41.1)	3631 (38.8)	<0.001
Stroke	8392 (38.3)	7963 (36.3)	<0.001	3901 (41.7)	3299 (35.3)	<0.001
Depression	1409 (6.4)	1167 (5.3)	<0.001	590 (6.3)	507 (5.4)	<0.001
CCI score (Mean ± SD)	2.26 ± 1.55	2.26 ± 1.55	1	2.33 ± 1.60	2.33 ± 1.60	1
Co-medications						
NSAIDs	13,085 (59.6)	11,095 (50.6)	<0.001	4519 (48.3)	3979 (42.6)	<0.001
Anti-hypertensives	9577 (43.7)	8248 (37.6)	<0.001	3439 (36.8)	3012 (32.2)	<0.001
Anti-diabetic agents	5332 (24.3)	5068 (23.1)	0.005	2736 (29.3)	2090 (22.4)	<0.001
Statins	2413 (10.9)	1842 (8.4)	0.003	919 (9.8)	798 (8.5)	0.025
Aspirin	5166 (23.5)	4887 (22.3)	0.002	2078 (22.2)	1578 (16.9)	<0.001
Anti-depressants	1074 (4.9)	775 (3.5)	<0.001	384 (4.1)	294 (3.1)	0.025
No. annual outpatient visits (Mean ± SD)	27.04 ± 18.78	20.87 ± 16.57	<0.001	22.32 ± 18.70	20.35 ± 16.68	<0.001

CAD, coronary artery disease; CCI, Charlson comorbidity index; SD, Standard deviation; GERD, gastroesophageal reflux disease; H2RA, histamine 2 receptor antagonist; NSAIDs, non-steroid anti-inflammatory drugs; PPI, proton pump inhibitor.

**Table 2:** Risk of exposure to acid suppressants for dementia development.

Exposure to Acid Suppressants	No. of Subjects	Dementia Development		Adjusted HR * (95% CI)
		No. of Cases	Incidence Rate (per 1000)	
H2RA non-users	21,939	4265	19.69	1.00 (Reference)
H2RA users	21,939	4743	22.71	1.84 (1.49–2.20)
cDDD (ref: H2RA non-users)				
Q_1_ (1–30)	15,057	3116	22.11	1.69 (1.23–1.86)
Q_2_ (31–180)	5834	1362	23.77	1.85 (1.46–2.24)
Q_3_ (181–365)	735	175	23.63	1.82 (1.50–2.36)
Q_4_ (>365)	313	90	28.62	1.96 (1.63–2.47)
				*p*_trend_ < 0.001
PPIs non-users	9348	1847	20.65	1.00 (Reference)
PPIs users	9348	1873	24.28	1.42 (1.07–1.84)
cDDD (ref: PPI non-users)				
Q_1_ (1–30)	4838	799	21.13	1.09 (0.91–1.37)
Q_2_ (31–180)	3924	904	27.34	1.59 (1.19–1.89)
Q_3_ (181–365)	438	119	30.54	1.82 (1.22–2.13)
Q_4_ (>365)	148	51	38.44	2.02 (1.43–2.31)
				*p*_trend_ < 0.001

CDDD, cumulative defined daily dose; CI, confidence interval; H2RA, histamine 2 receptor antagonist; HR, hazard ratio; PPI, proton pump inhibitor; ref, the reference group; * Hazard ratios were adjusted for sex, age, index year, Charlson comorbidity index, numbers of annual outpatient visits, and use of co-medications, including non-steroidal anti-inflammatory drugs and aspirin, and mutually adjusted for use of H2RAs and PPIs; The reference group was H2RA non-users or PPI non-users; Q_1_, Q_2_, Q_3_, and Q_4_ were quartiles of cDDD of H2RA use or PPI use.

**Table 3:** Risk of dementia associated with exposure to histamine 2 receptor antagonists (H2RAs) stratified by the use of proton pump inhibitors (PPIs).

Use of Acid Suppressants	No. of Subjects	Dementia Development		Adjusted HR * (95% CI)
		No. of Cases (%)	Incidence Rate (per 1000)	
PPI users				
H2RA non-users	9036	1662 (18.4)	21.40	1.00 (Reference)
H2RA users	4994	865 (17.3)	17.56	0.92 (0.85–1.06)
PPI non-users				
H2RA non-users	23,277	4570 (19.6)	19.99	1.00 (Reference)
H2RA users	15,534	3531 (22.7)	23.97	1.86 (1.37–2.02)
H2RA users				
PPI non-users	15,534	3531 (22.7)	23.97	1.00 (Reference)
PPI users	4994	865 (17.3)	17.56	0.76 (0.61–1.83)
H2RA non-users				
PPI non-users	23,277	4570 (19.6)	19.99	1.00 (Reference)
PPI users	9036	1662 (18.4)	21.40	1.23 (1.08–2.59)

* Hazard ratios were adjusted for sex, age, index year, Charlson comorbidity index, use of co-medications, including non-steroidal anti-inflammatory drugs and aspirin, and numbers of annual outpatient visits.

**Table 4:** Association between acid suppressant combination and dementia risk.

Acid Suppressant Combination Use	No. of Subjects	Dementia Development		Adjusted HR * (95% CI)
		No. of Cases	Incidence Rate (per 1000)	
Neither	23,277	4570	19.99	1.00 (Reference)
H2RA only	15,534	3531	23.97	1.85 (1.26–2.16)
PPI only	9036	1662	21.40	1.64 (1.14–1.92)
Concurrent use	4994	865	20.57	1.23 (1.07–1.98)

* CI, confidence interval; H2RA, histamine 2 receptor antagonist; HR, hazard ratio; PPI, proton pump inhibitor. ^*^ Hazard ratios were adjusted for sex, age, index year, Charlson comorbidity index, use of co-medications, including non-steroidal anti-inflammatory drugs and aspirin, and numbers of annual outpatient visits.
